# BDNF and Cognitive Function in Chilean Schizophrenic Patients

**DOI:** 10.3390/ijms241310569

**Published:** 2023-06-24

**Authors:** Rodrigo R. Nieto, Hernán Silva, Alejandra Armijo, Rubén Nachar, Alfonso González, Carmen Paz Castañeda, Cristián Montes, Manuel Kukuljan

**Affiliations:** 1 Clínica Psiquiátrica Universitaria, Hospital Clínico de la Universidad de Chile, Universidad de Chile, Santiago 8380453, Chile; 2 Departamento de Psiquiatría y Salud Mental Norte, Facultad de Medicina, Universidad de Chile, Santiago 8380453, Chile; 3 Departamento de Neurociencias, Facultad de Medicina, Universidad de Chile, Santiago 8380453, Chile; 4 Laboratorio de Neurobiología Celular y Molecular, Facultad de Medicina, Universidad de Chile, Santiago 8380453, Chile; 5 Hospital Dr. José Horwitz Barak, Servicio de Salud Metropolitano Norte, Santiago 8431621, Chile; 6 Escuela de Medicina, Universidad Finis Terrae, Santiago 7501015, Chile

**Keywords:** brain-derived neurotrophic factor, neurotrophins, schizophrenia, cognition, Montreal Cognitive Assessment (MoCA), cognitive impairment, cognition biomarker, BDNF plasma levels

## Abstract

Despite cognitive symptoms being very important in schizophrenia, not every schizophrenic patient has a significant cognitive deficit. The molecular mechanisms underlying the different degrees of cognitive functioning in schizophrenic patients are not sufficiently understood. We studied the relation between brain-derived neurotrophic factor (BDNF) and cognitive functioning in two groups of schizophrenic patients with different cognitive statuses. According to the Montreal Cognitive Assessment (MoCA) results, the schizophrenic patients were classified into two subgroups: normal cognition (26 or more) and cognitive deficit (25 or less). We measured their plasma BDNF levels using ELISAs. The statistical analyses were performed using Spearman’s Rho and Kruskal–Wallis tests. We found a statistically significant positive correlation between the plasma BDNF levels and MoCA score (*p* = 0.04) in the subgroup of schizophrenic patients with a cognitive deficit (*n* = 29). However, this correlation was not observed in the patients with normal cognition (*n* = 11) and was not observed in the total patient group (*n* = 40). These results support a significant role for BDNF in the cognitive functioning of schizophrenics with some degree of cognitive deficit, but suggest that BDNF may not be crucial in patients with a normal cognitive status. These findings provide information about the molecular basis underlying cognitive deficits in this illness.

## 1. Introduction

Schizophrenia is a severe psychiatric disorder that has a significant impact on patients, their families, and society [1]. Although there is heterogeneity in their clinical presentation, schizophrenic symptoms can be grouped into positive, such as hallucinations and delusions, negative, such as anhedonia and apathy, and cognitive symptoms [2]. The latter include deficits of varying degrees in all cognitive domains, including memory, processing speed, attention, and executive functions, and have been consistently demonstrated in different studies across several geographic regions [3]. In recent decades, evidence has accumulated indicating that cognitive symptoms constitute the most important predictors of community functioning, particularly in schizophrenia patients, as cognitive deficits may prevent these patients from attaining optimal adaptation and therefore act as neurocognitive rate-limiting factors for functional outcomes [4,5].

Despite cognitive symptoms being very important in schizophrenia, not every schizophrenic patient has a significant cognitive deficit, and individual patients´ cognitive deficits may vary significantly [6,7]. The molecular mechanisms underlying the different degrees of cognitive functioning in schizophrenic patients are not sufficiently understood. In order to address this issue, several lines of research have aimed to find biomarkers for cognition in schizophrenia. Among other potential candidates, brain-derived neurotrophic factor (BDNF) has been studied considering its roles in neurodevelopment, neuroprotection, synaptic plasticity, learning, and memory [8,9,10].

Neurotrophins such as BDNF regulate synapse maturation at the morphological, molecular, and functional levels. The role of BDNF in synapses is crucial not only during development, but also for synaptic plasticity in adults [11], and can produce long-term changes in the functionality of adult neurons through changes in gene expression. However, the cytoplasmic effectors activated by neurotrophins also exert a wide range of more rapid actions, including the modulation of neuronal excitability and synaptic transmission [12,13]. BDNF secretion is required for long-term potentiation (LTP) and long-term depression (LTD), which are the molecular mechanisms underlying learning and memory [12,14]. An increased expression of BDNF can have a positive effect on the generation of LTP and memory [15]. In animal models, BDNF mutant mice show learning deficits and other hippocampal-dependent altered cognitive features [16]. In relation to long-term memory, is noteworthy that BDNF is sufficient for inducing the transformation of early-phase LTP to late-phase LTP, and an inhibition of the signaling of BDNF in animal models also alters long-term memory [17].

Evidence has increasingly suggested that schizophrenia is a subtle disorder of brain development [18], and alterations in BDNF could lead to altered brain development, with inappropriate variations in the cortical circuitry and synaptic transmission in the developing brain, which could then translate into the neural dysfunction underlying schizophrenia [19,20]. The BDNF levels in the peripheral blood are reduced in schizophrenic patients compared to healthy control subjects, but there is a considerable and unexplained heterogeneity between different studies [21]. BDNF levels have been found to be positively correlated with cognitive function in most studies, both in schizophrenic patients and healthy subjects [22,23,24], but some have reported a negative correlation between BDNF levels and some cognitive domains in healthy subjects [25]. Meta-analyses performed so far have not been able to robustly establish an association between BDNF and neurocognition, mainly due to inconsistent results among different studies [26,27].

BDNF’s ability to cross the blood–brain barrier suggests that the BDNF levels measured in the peripheral blood may reflect its levels in the brain [28]. The relation between peripheral and brain BDNF was demonstrated by a Pillai et al. [29] in a study that showed parallel changes in the BDNF levels in the plasma and CSF of patients with schizophrenia, indicating that these plasma BDNF levels reflect the brain changes in BDNF levels. Therefore, measuring plasma BDNF levels is a valid model for studying the role of neurotrophins in schizophrenia patients.

We hypothesized that one source of the different results between studies is the heterogeneity due to the different degrees of cognitive deficit among patients. Therefore, we aimed to study the relation between plasma BDNF levels and cognition in two separate groups of schizophrenic patients with different cognitive statuses: with and without a cognitive deficit.

## 2. Results

We recruited a sample of 40 Chilean patients with a diagnosis of schizophrenia undergoing treatment with at least one second-generation antipsychotic, who were clinically stable, in order to perform neurocognitive testing. Additionally, a sample of 14 healthy subjects was recruited for comparison. The main characteristics of both patients and the healthy subjects group are in Table 1. All the participants gave written informed consent in a consent form approved by the local IRB.

### 2.1. BDNF Plasma Levels

**Figure 1:**
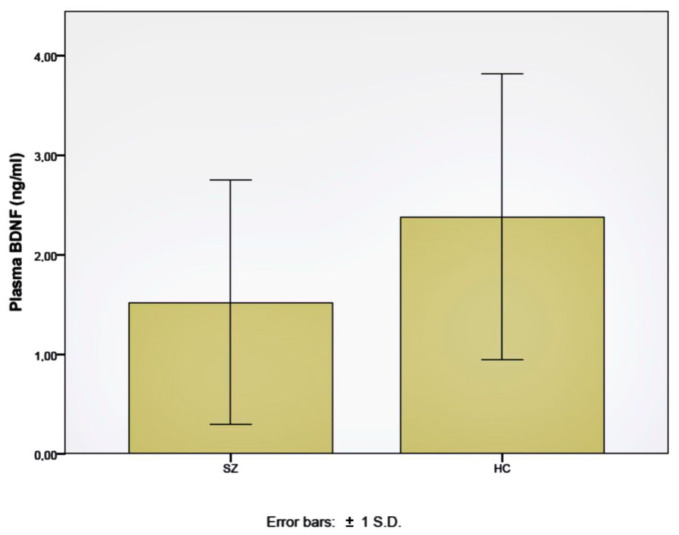
Plasma BDNF levels in schizophrenia patients (SZ) and healthy control subjects (HC).

The BDNF plasma levels were measured using ELISAs in 40 patients with diagnoses of schizophrenia and 14 subjects from the control group. An average value of 1.52 ng/mL was obtained for the patients and 2.38 for the control group subjects, with standard deviations of 1.22 and 1.43, respectively (Figure 1). The BDNF plasma levels were significantly lower in the patients than in the healthy control subjects, according to the Kruskal–Wallis test (*p* = 0.04).

### 2.2. Cognitive Assessments

**Figure 2:**
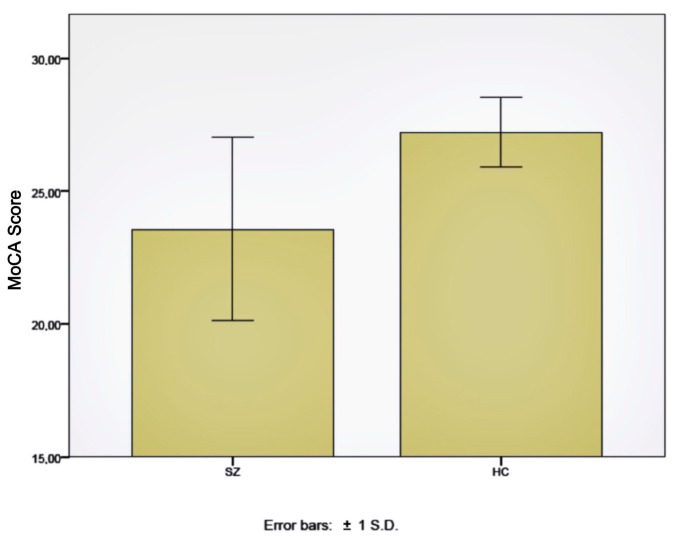
Montreal Cognitive Assessment (MoCA) score in schizophrenia patients (SZ) and healthy control subjects (HC).

Neurocognitive evaluations were carried out using the Montreal Cognitive Assessment (MoCA) [30] in all 40 patients, with an average score of 23.6 points and a standard deviation of 3.4 points. In total, 70% of these patients (*n* = 28) had a result compatible with a cognitive deficit, that is, a score of less than 26 points. In the healthy control subjects group, the average score was 27.3 points, with a standard deviation of 1.8. Only one of these subjects obtained a result compatible with a cognitive deficit, in this case, 25 points. The MoCA scores were significantly lower in the schizophrenia patients than the healthy subjects (*p* < 0.01) (Figure 2), and the proportion of subjects with normal MoCA and MoCA deficit scores were significantly different between both groups, according to the Kruskal–Wallis test (*p* < 0.001).

### 2.3. Correlation between BDNF Levels and Cognitive Assessments

**Figure 3:**
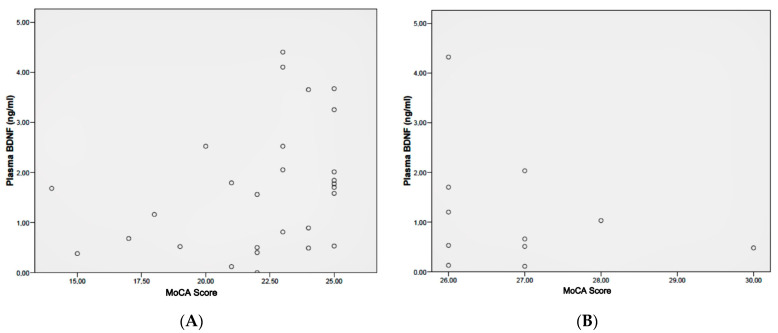
Correlation between plasma BDNF levels and Montreal Cognitive Assessment (MoCA) score in: (**A**) schizophrenia patients with cognitive deficit according to MoCA (i.e., lower than 26 points), and (**B**) schizophrenia patients with a normal MoCA score (i.e., 26 or higher).

In the complete group of patients with schizophrenia, the BDNF plasma levels were not significantly correlated with the MoCA scores (*p* = 0.90), according to the Spearman Rho test. However, if we split the schizophrenia patients group into two subgroups according to their cognitive statuses (normal MoCA score vs. MoCA deficit score), we found a statistically significant positive correlation between the BDNF plasma levels and MoCA scores in the subgroup of patients with a cognitive deficit, according to the Spearman Rho test (*p* = 0.04). This correlation was not found in the subgroup of patients with a normal MoCA score (*p* = 0.41). (Figure 3A,B). This correlation was also not found in the healthy subjects control group (*p* = 0.59). Overall, the BDNF plasma levels were not significantly different when comparing the patients with a normal cognition to those who had a cognitive deficit (*p* = 0.22).

## 3. Discussion

This study addresses the issue of studying the role of BDNF in different groups of schizophrenic patients in a way that has not been done before. Our results support a significant role for BDNF in the cognitive functioning of schizophrenics with some degree of cognitive deficit, but suggest that BDNF may not be crucial in patients with a normal cognitive status. This should be taken into account in future studies that aim to understand the relation between BDNF and cognitive symptoms in schizophrenic patients.

Cognitive symptoms are a central manifestation of schizophrenia and are significantly related to patients’ community functioning. However, individual schizophrenic patients have different degrees of cognitive impairment, and although most of them have some degree of cognitive deficit, some patients achieve normal results in neuropsychological testing [6,7]. Previous studies have found a positive correlation between the peripheral BDNF levels in both schizophrenic patients and healthy subjects, but a negative correlation between BDNF levels and cognitive function has been reported in healthy subjects [22,23,24,25].

Considering this, we aimed to study the relation between BDNF and cognitive functioning in two groups of schizophrenic patients with different cognitive statuses, finding a positive, statistically significant correlation (*p* = 0.04) between the plasma BDNF levels and MoCA scores in the group of patients with schizophrenia who presented a cognitive deficit, according to the neurocognitive evaluation (*n* = 29). This is consistent with the role of BDNF in long-term potentiation (LTP), which is a critical molecular mechanism underlying learning and memory [12,13,14,15] and in synaptic plasticity [11,12,13]. However, this correlation was not observed in the patients with normal cognition (*n* = 11) and also it was not observed in the total patient group (*n* = 40). These results support a role for BDNF in the cognitive functioning of schizophrenic patients, but this role seems to be particularly relevant in patients with some degree of cognitive deficit and may not be crucial in patients with normal cognition. Possibly, in patients with normal cognition, higher BDNF levels could be of no additional benefit, similar to what could be happening in healthy subjects.

We also found lower BDNF plasma levels (*p* = 0.04) in our sample of Chilean schizophrenic patients in comparison to those in local healthy subjects. This is consistent with what was described in the meta-analysis by Green et al. [21]. In this regard, this could be considered another significant contribution of our research, since it allows for the addition of data from an under-represented continent for future meta-analyses that seek to compare the BDNF levels in schizophrenic patients and healthy subjects.

Alterations in neurotrophic factors such as BDNF may contribute to altered brain development, a lack of synaptic connectivity, and problems in neuroplasticity. They may explain, at least in part, some of the morphological and neurochemical abnormalities that have been found in the brains of patients with schizophrenia [19,31,32]. A recent meta-analysis provided further evidence of the associations between brain volume alterations in schizophrenia and BDNF peripheral levels [33]. The role of BDNF in learning and memory has been demonstrated in cellular and molecular studies, where BDNF secretion has been shown to be required for long-term potentiation (LTP) and long-term depression (LTD), which are the molecular mechanisms underlying learning and memory [12,13,14]. In animal models, the role of BDNF in cognition has been evaluated in studies with BDNF mutant mice showing learning deficits and impaired hippocampal-dependent pattern discrimination [16,34]. In human subjects, low peripheral BDNF levels are associated with a reduction in hippocampal volume at the onset of schizophrenia [33,35].

Several limitations need to be addressed. First, the clinical heterogeneity in our sample. Most of the patients had a relatively short evolution time of the illness, considering that 85% (*n* = 34) met the criteria for recent-onset schizophrenia, which is defined as less than five years from the onset of positive symptoms. This could also be considered as a significant contribution of this study, since previous studies have focused on chronic schizophrenia patient populations. Regarding the cognitive assessments, MoCA is a valid screening tool for mild cognitive impairment and was not designed for patients diagnosed with schizophrenia, unlike cognitive batteries such as the MATRICS Consensus Cognitive Battery (MCCB) [36]. However, unlike MCCB, the fact that MoCA has a clear cut-off score for determining whether a person has at least a mild cognitive deficit seemed appropriate for the purpose of this research; it has been described as a useful tool for screening cognitive impairment in patients with schizophrenia [37]. Finally, with regard to the measurement of the plasma BDNF levels, it is necessary to consider several potential aspects of variability in the results. Variations in the pre-analytical conditions of the plasma samples, particularly with respect to temperature, may have altered the results [38]. In order to control this source of variation, we decided to transport the samples to the laboratory at 4 °C. The effects of physical exercise and menstrual cycles were not appropriately controlled and must be acknowledged as additional limitations. Another relevant aspect is the relationship with circadian variations throughout the day. To control this source of variation, the samples were taken at approximately the same time of day (around noon).

In conclusion, in this study, plasma BDNF was positively correlated with cognitive symptoms only in the subgroup of patients with a cognitive deficit, but not in the subgroup of patients with a normal cognitive score, nor in the control group of healthy subjects. This may be one of the reasons why several previous studies that aimed to correlate BDNF levels and cognition in schizophrenic patients have found inconsistent results. Therefore, research on BDNF as a biomarker of cognition in schizophrenia needs to be tailored to specific subgroups of patients, including stratification according to different degrees of a patient´s cognitive impairment. Future treatment studies aiming to improve cognitive symptoms, both pharmacological and non-pharmacological studies, should consider this to guide potential therapeutic strategies for the improvement of cognitive symptoms [39]. In a broader way, a deeper understanding of BDNF’s role in schizophrenia could inform the development of BDNF-based strategies for the prevention and treatment of this disorder [40]. This approach will provide additional information about the molecular mechanisms underlying the cognitive dysfunction in schizophrenia and is required to be complemented with the study of other potentially relevant biomarkers.

## 4. Materials and Methods

### 4.1. Subjects

Forty Chilean patients with schizophrenia (DSM-IV) were recruited from both the University of Chile Psychiatric Clinic and the public Howritz Barak Psychiatric Hospital, in Santiago, Chile. In a non-randomized manner, we evaluated the first 40 patients that fulfilled the inclusion criteria and did not meet the exclusion criteria, and accepted them to participate in our study. The inclusion criteria required an SCID-I diagnosis of schizophrenia and less than 12 years since their first psychotic episode. The exclusion criteria were the active consumption of addictive drugs and a significant medical or neurological comorbidity. All the study participants provided written informed consent after the procedure had been fully explained, as approved by the local Ethics Committee (protocol code OAIC 497/11).

### 4.2. Clinical and Cognitive Assessments

The clinical symptoms were assessed using the Positive and Negative Syndrome Scale (PANSS). Relevant demographical and clinical information was recollected, such as age, gender, years of education, age of onset, duration of illness, and treatment characteristics, including the mean dose of chlorpromazine equivalents. The cognitive functioning of the patients was evaluated using the Montreal Cognitive Assessment (MoCA).

### 4.3. BDNF Levels Measurements

The plasma samples of the participants were collected on the same day that the clinical and cognitive evaluations were performed and stored at −80 °C until they were assayed. The BDNF levels were measured with a human BDNF ELISA Kit, which was performed as instructed by the manufacturer (R&D Systems, Minneapolis, MN, USA).

### 4.4. Statistical Analysis

To explore the normal distribution of the variables, we performed a Shapiro–Wilk test. According to the result, for the subsequent analyses, we used non-parametric tests. To study the differences between the groups we performed a Kruskal–Wallis test. For the correlation analyses, we performed Spearman’s Rho tests, with a bilateral significance test.

## Figures and Tables

**Table 1:** Main characteristics of schizophrenia patients and healthy subjects control group.

	Schizophrenia	Healthy Subjects
*n*	40	14
Gender (M/F)	34/6	10/4
Age	22.7 (±4.7)	23.8 (±3.5)
Years of education	12.4 (±2.2)	15.9 (±2.1)
Age of onset	20.1 (±3.2)	-
Years since onset	2.7 (±3.2)	-
Ever hospitalized	19/40 (47.5%)	-
SGA treatment	37 (92.5%)	-
Antidepressants	9 (22.5%)	-
PANSS score	70.04 (±31.5)	-
BDI score	11.45 (±10.3)	-
CGI score	2.84 (±1.2)	-

The main characteristics of schizophrenia patients and healthy subjects control group are shown here, presenting mean ± standard deviation for age, years of education, years since onset, PANSS score, BDI score, and CGI score, and the number of subjects and % of the group for use of second-generation antipsychotics (SGA), antidepressants, and if the patients had been hospitalized.

## Data Availability

The data presented in this study are available on request from the corresponding author. The data are not publicly available due to ethical restrictions, since no permission was asked in the informed consent for sharing data.
